# Structure and dynamics of catalytically competent but labile paramagnetic metal-hydrides: the Ti(iii)-H in homogeneous olefin polymerization†

**DOI:** 10.1039/d0sc04967k

**Published:** 2020-09-24

**Authors:** Enrico Salvadori, Mario Chiesa, Antonio Buonerba, Alfonso Grassi

**Affiliations:** Department of Chemistry, University of Turin Via Pietro Giuria 7 Torino 10125 Italy mario.chiesa@unito.it; Dipartimento di Chimica e Biologia, Università degli Studi di Salerno Via Giovanni Paolo II, 132 I-84084 Fisciano SA Italy

## Abstract

Metal hydride complexes find widespread application in catalysis and their properties are often understood on the basis of the available crystal structures. However, some catalytically relevant metal hydrides are only spontaneously formed *in situ*, cannot be isolated in large quantities or crystallised and their structure is therefore ill defined. One such example is the paramagnetic Ti(iii)-hydride involved in homogeneous Ziegler–Natta catalysis, formed upon activation of CpTi(iv)Cl_3_ with modified methylalumoxane (MMAO). In this contribution, through a combined use of electron paramagnetic resonance (EPR), electron-nuclear double resonance (ENDOR) and hyperfine sublevel correlation (HYSCORE) spectroscopies we identify the nature of the ligands, their bonding interaction and the extent of the spin distribution. From the data, an atomistic and electronic model is proposed, which supports the presence of a self-assembled ion pair between a cationic terminal Ti-hydride and an aluminate anion, with a hydrodynamic radius of *ca.* 16 Å.

## Abstract 2

Catalytically competent titanium-hydrides are reactive and difficult to isolate. We use EPR spectroscopy to define the electronic and geometrical structures as well as dynamics of an open-shell Ti-H active in syndiospecific olefin polymerization.

## Graphical abstract

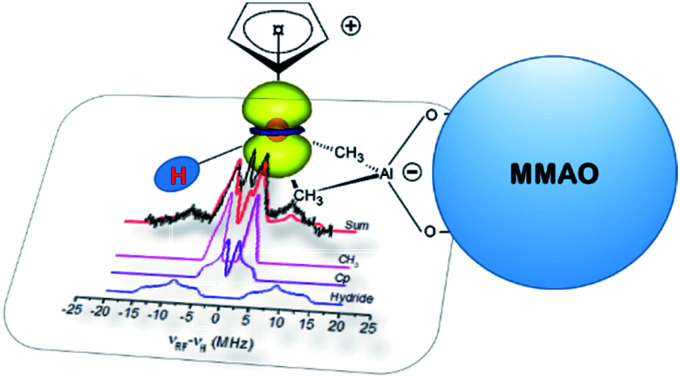

## Introduction

Transition metal hydrides play crucial roles in many catalytic processes of importance in biology, materials science and industrial chemistry. Indeed, M–H bonds are involved in the biosynthesis of dihydrogen by hydrogenase,^1^ in nitrogen fixation by MoFe cofactor^2^ as well as in different catalytic reactions of industrial relevance such as hydrogenation of multiple carbon–carbon and carbon–heteroatom bonds,^3^ hydrocyanation,^4^ hydroformylation,^5^ hydrosilylation^6^ and in olefin isomerisation and polymerisation.^7,8^ The structure and chemistry of metal-hydride species involved in homogeneous catalysis has been comprehensively reviewed by Butler and Crimmin.^9^ However, only recently thorough characterisation of paramagnetic systems has gained momentum.^10^ Focusing on titanium, Ti-hydride complexes usually belong to the class of heterobimetallic bridged hydrides with general formula Ti-H-M (M = Mg, Zn, Al, Ga), these are active in a number of relevant synthetic reactions^11^ and are stable enough to be crystallized. Ti/Al heterobimetallic hydrides are usually obtained by salt-metathesis from Ti(iv) precursors whereby the formation of the Ti-H-Al motif often leads to the Ti(iii) paramagnetic state. Such open-shell hydrides are notoriously challenging to characterise in particular when formed *in situ* owing to their short-lived and labile nature, which hampers standard structural characterisation. One such example is the Ti(iii)-hydride complex formed during the activation reaction of titanium half-metallocenes with methylalumoxane (MAO) which is the subject of this work. In the early 1980s, the development of single site homogenous olefin polymerization catalysts based on group 4 metallocenes or half-metallocenes activated with MAO pivoted the mechanistic understanding of various features of olefins polymerization. To date, this reaction is one of the most investigated and well-understood processes where most mechanistic details concerning the catalytic cycle are well-defined and modelled.^12–14^ MAO is an alkyl aluminum compound of general formula [–Al(CH_3_)O–]_*n*_ coordinating a variable amount of AlMe_3_ and featuring three-dimensional cage-like cluster structures with tetrahedral aluminum centers bridged by three-coordinate oxygen atoms.^15–17^ In modified MAO (MMAO), bulkier isobutyl or isooctyl groups partially replace the methyl groups in the MAO structure.^18,19^ During the *in situ* activation of group 4 metallocenes, MAO/MMAO acts as an alkylating agent leading to a metal–alkyl bond, *via* ligand exchange reactions. Moreover, in the case of titanocenes and half-titanocenes, the Ti(iv) pre-catalysts can be readily reduced to lower oxidation states, namely Ti(iii) and Ti(ii), due to the lower redox potential of this metal as compared to Zr and Hf. This reduction process is thought to be at the origin of the extremely high polymerization activity of the CpTiX_3_ catalysts (Cp = μ^5^-C_5_H_5_; X= halide; alkyl) in syndiospecific styrene polymerization,^20–23^ chemoselective polymerization of conjugated dienes^24–26^ and the synthesis of stereoregular styrene-butadiene^27,28^ copolymers where the role of the Ti(iii) species as active catalysts has been firmly assessed.^29^ The formation of a number of open-shell Ti(iii) species upon MAO/MMAO activation was demonstrated through conventional continuous wave (CW) electron paramagnetic resonance (EPR) spectroscopy in solution.^30–32^ Furthermore, quantitative ^1^H NMR analysis of the syndiotactic polystyrene produced under quasi living polymerization conditions (polydispersity index (PDI) = 1.3–1.6) by these catalysts demonstrated that Ti(iii)-H species are involved in the initiation reaction and that a stationary concentration (∼20% of the paramagnetic fraction) is produced under polymerization conditions through a reversible insertion–elimination of the growing polymer chain.^33^

Despite their relevance, the chemical structure of such open-shell Ti(iii) hydride complexes and the origin of these species are still an open question of great interest in MAO activated half-titanocene catalysis. NMR spectroscopy has been instrumental in elucidating the activation and the structure of Ti(iv) cationic complexes and their stabilization through formation of ion pairs formed upon MAO/MMAO activators.^34–37^ Similar detailed spectroscopic studies of the corresponding Ti(iii) open-shell complexes are completely lacking. EPR is especially suited for this task as the magnetic hyperfine couplings between the electron spin and the surrounding magnetically active nuclei encode geometrical information such as the distance between the magnetic nuclei and the electron spin-carrying centre and their relative orientation. So far only conventional X-band CW EPR techniques have been used to characterize the paramagnetic products generated upon activation of the precursors, providing key insight into the reaction mechanism.^30–32^ However, pulsed EPR offers a whole arsenal of specific experiments (ENDOR, ESEEM, HYSCORE) capable of investigating the local coordination environment of paramagnetic species,^38–41^ affording resolution comparable to that of advanced NMR techniques.

In this contribution we employ X- and Q-band EPR spectroscopy to elucidate the electronic and geometrical structure of Ti(iii) species obtained by reaction of CpTiCl_3_ with MMAO. We provide compelling evidence for a terminal Ti-H bond and for the presence of a ^27^Al in the second coordination sphere of the Ti(iii) cationic complex. This is the first detailed structural description of the metal hydride formed *in situ* upon reductive activation of a titanium half-metallocene precursor, which is stabilized by the formation of an ion pair with the alumoxane moiety.

## Results and discussion

### Solution X-band CW EPR

**Fig. 1:**
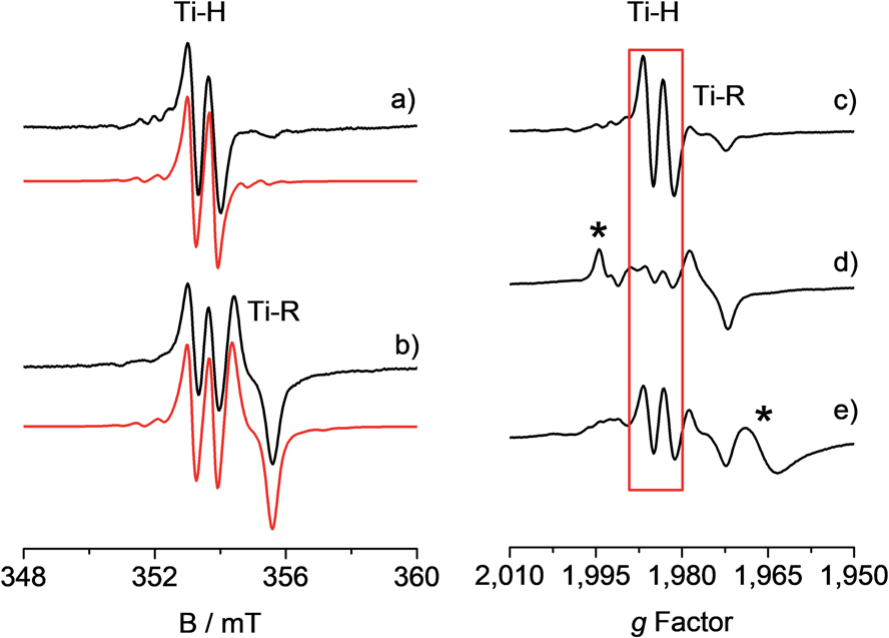
Experimental (black) and simulated (red) X-band EPR spectra of CpTiCl_3_ in presence of MMAO ([Ti] = 6.0 mM; Al/Ti molar ratio = 250) recorded (a) after 5 minutes and (b) after 95 minutes from the mixing of the reagents. The simulated spectrum in b is the sum of the Ti-H and Ti-R components, with relative weight 30 : 70. On the right hand side the X-band CW-EPR spectra recorded during a polymerization reaction performed *in situ* in the EPR cell are shown on the *g*-scale: (c) activated catalyst (CpTiCl_3_ in presence of MMAO, [Ti] = 6.0 mM; Al/Ti molar ratio = 250); (d) after 10 minutes of reaction with styrene; (e) after 45 minutes of reaction and heating at 50 °C. The Ti-H signal is highlighted in red while asterisks indicate extra species formed during the polymerisation reaction. Experimental conditions: temperature = 298 K, mw_frequency_ = 9.8 GHz, mw_power_ = 10 mW. Simulation parameters are summarized in Table 1.

The toluene solution of the CpTiCl_3_ precursor is EPR silent, as expected for a diamagnetic Ti(iv) system. When such solution was treated with MMAO (see also Fig. S1 in the ESI†) under argon atmosphere, the resulting reaction mixture ([Ti] = 6.0 mM; Al/Ti molar ratio = 250; 25 °C) readily turned from a red to a brown colour. The corresponding room temperature X-band EPR CW spectra recorded at different reaction times after preparation and their relative simulations are given in Fig. 1. The 5 minute spectrum (Fig. 1a) consists of a doublet centred at *g*_iso_ = 1.9917 ± 0.0002 – characteristic of a Ti(iii) ion – and separated by 0.65 mT. Minor satellite peaks are visible at either side of the spectrum and account for ^47,49^Ti (^47^Ti: 7.44%, *I* = 5/2; ^49^Ti: 5.41%, *I* = 7/2) hyperfine couplings, if a |*a*_iso_^Ti^| = 17.5 ± 0.2 MHz is imposed in the simulation. This |*a*_iso_^Ti^| value is consistent with literature reports on similar systems^42^ and corresponds to a spin density at Ti of ∼95%. Given that ^1^H and ^35,37^Cl are the only magnetically active nuclei present in significant natural abundances and based on ^1^H ENDOR experiments (*vide infra*), the doubled splitting is assigned to the hyperfine coupling of the unpaired electron with a ^1^H nucleus with |*a*_iso_^H^| = 18.16 ± 0.20 MHz, corresponding to a spin density at the proton of 1.3% (see ESI†). These values are in agreement with previous studies on related systems.^32–34^ When the sample is left to evolve at room temperature, the intensity of the doublet spectrum smoothly decreases over time as a second species gradually appears, while the total paramagnetic fraction stays constant. Besides the already discussed doublet, the 95 minute spectrum (Fig. 1b and S2 in the ESI†) shows this second species characterized by a *g*_iso_ = 1.9821 ± 0.0002 and line shape with a characteristic inflection point in the middle that can be simulated by imposing a ^27^Al hyperfine coupling of |*a*_iso_^Al^| = 6.2 ± 0.3 MHz (^27^Al: 100%, *I* = 5/2). Both these signals have been previously attributed^32,43^ to a Ti-hydride adduct (Ti-H hereafter) and a Ti-alkyl adduct (Ti-R) stabilized by MAO.

One crucial point at this stage is the demonstration of the catalytic relevance of the Ti-H species. To this end, X-band CW-EPR spectra were recorded at different stages of the polymerization reaction with styrene, which was chosen as a benchmark substrate. The EPR spectrum of the activated sample is shown in Fig. 1c, it displays the characteristic doublet of the Ti-H and a small fraction of the Ti-R species. Polymerization of styrene was initiated by adding 0.3 mL of anhydrous styrene to the catalyst. In the EPR spectrum recorded after 10 minutes from the mixing (Fig. 1d) a drastic decrease of the Ti-H signal was observed as the polymer started forming in the EPR tube. After 45 min of reaction followed by heating at about 50 °C, the Ti-H signal grows again (Fig. 1e) indicating that the Ti-H species is restored under these conditions. The decrease of the Ti-H signal during the initial stages of the polymerization points to a direct involvement in the initiation reaction, whereby a styrene monomer is inserted in the Ti-H bond producing a Ti-R species. On the other hand, restoring of the Ti-H signal intensity at high polymerization temperature can be explained considering the generation of Ti-H species in the course of the polymerization reaction *via* β-hydride transfer from the last monomer unit of the growing polymer chain (see Scheme 2). The oscillating nature of the Ti-H signal thus firmly demonstrates that this species is actively involved in the catalytic cycle, participating in both initiation and termination reactions.^33^ In the following, we use pulsed EPR techniques to determine the electronic and geometrical structure of the Ti-H species.

### Solid state Q-band CW EPR

**Fig. 2:**
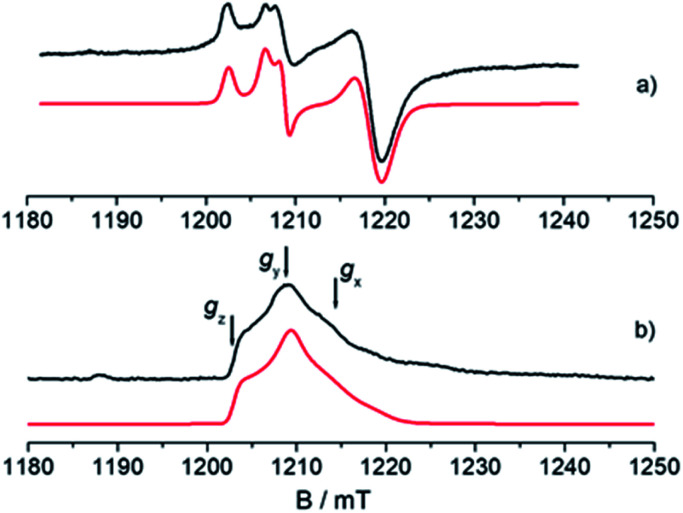
Experimental (black) and simulated (red) Q-band EPR spectra of CpTiCl_3_ after reaction with MMAO. (a) CW spectrum recorded with experimental parameters: temperature = 50 K, mw_frequency_ = 33.7 GHz, mw_power_ = 1 mW. The simulated spectrum is the sum of the Ti-H and Ti-R components, with relative weight 35 : 65. (b) Two-pulse ESE-EPR spectrum recorded with experimental parameters: temperature = 50 K, mw_frequency_ = 33.7 GHz, interpulse delay = 200 ns. The simulated spectrum only accounts for the Ti-H component. Simulation parameters are summarized in Table 1. Arrows indicate the magnetic field settings at which Davies ENDOR and HYSCORE experiments were performed.

An understanding of the electronic ground state and the coordination geometry of the Ti(iii) ion can be obtained if the full anisotropy of the **g** tensor is resolved. To this end, Q-band CW spectroscopy at cryogenic temperature is advantageous as the suppression of molecular reorientation with respect to the applied magnetic field allows the resolution of the full **g** tensor, and the higher applied magnetic field permits greater accuracy in the determination of its principal values. Fig. 2 shows a representative experimental CW Q-band spectrum measured at 50 K and its simulation (see also Fig. S2 in the ESI†). The spectrum shows two partially overlapping signals, one with a rhombic **g** tensor [*g*_*x*_, *g*_*y*_, *g*_*z*_] = [1.9812, 1.9916, 2.0023] and the other with an almost axial **g** tensor [*g*_*x*_, *g*_*y*_, *g*_*z*_] = [1.9737, 1.9770, 1.9956]. On the basis of the *g*_iso_ measured at room temperature at X-band and the relationship *g*_iso_ = 1/3(*g*_*x*_ + *g*_*y*_ + *g*_*z*_), it can be concluded that the rhombic tensor belongs to the Ti-H signal, whereas the Ti-R species accounts for the nearly axial tensor. The largest principal *g* value (*g*_*z*_) of Ti-H corresponds, within the experimental error, to the free electron value (*g*_e_ = 2.0023) unambiguously indicating a singly occupied molecular orbital (SOMO) comprised predominantly of a Ti d_*z*^2^_ orbital. In this picture the directions of the principal *g* value *g*_*z*_ (2.0023) can be set along the cylindrical axis of the d_*z*^2^_ orbital, whereas the directions of *g*_*x*_ and *g*_*y*_ cannot be unequivocally determined at this stage.

**Scheme 1:**
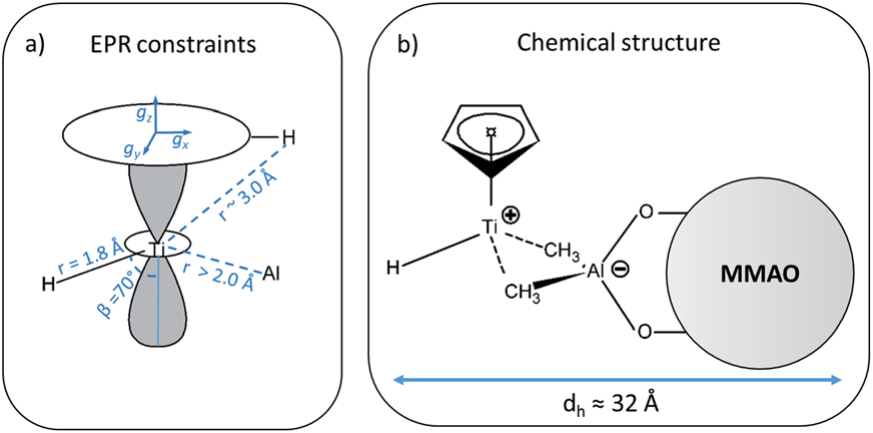
(a) Summary of all constraints and structural parameters derived from the analysis of the EPR experiments (for clarity, only one out of the five Cp protons is shown), dashed lines represent trough–space interaction, whereas continuous lines represent direct bonds; (b) chemical structure derived from the EPR data, dashed lines represent the ion pair interaction between the [CpTi(iii)H]^+^ cation and the MMAO anion. The hydrodynamic diameter (*d*_h_) of the ion pair is also reported.

### Electron Spin Echo (ESE)-detected EPR

Further information on the electronic and geometrical structure of the paramagnetic Ti(iii) complexes can be obtained through pulse EPR measurements.

The Q-band ESE-detected EPR spectrum recorded at 50 K is shown in Fig. 2b. The echo detected EPR signal corresponds to the absorption of the CW EPR spectrum, however, inspection of the signal in Fig. 2b reveals that the axial component resonating at 1220 mT is missing in the ESE spectrum. This clearly indicates that such species are characterized by fast relaxation times and are filtered out in the ESE spectrum, which can be well simulated considering only the parameters relative to the Ti-H species (Fig. 2b and Table 1). The simulation accounts for 90% of the observed signal and displays a poorer agreement with the experimental spectrum only in the high field region where the major contribution of the Ti-R species would be expected. Attempts to recover the Ti-R component by lowering the temperature at 20 K were not successful. The very different relaxation dynamics of Ti-H and Ti-R allow the selective study of the former *via* hyperfine spectroscopy in order to define the coordination environment of the Ti(iii) centre.

**Table 1:** Summary of the EPR parameters for Ti-H and Ti-R derived in this work, errors were estimated from spectral simulations. All hyperfine and nuclear quadrupole interactions are given in units of MHz, while angles are in degrees

	Ti-H	Ti-R
*g*_*x*_, *g*_*y*_, *g*_*z*_	[1.9812, 1.9916, 2.0023] ± 0.0002	[1.9737, 1.9770, 1.9956] ± 0.0002
*g*_iso_	1.9917 ± 0.0002	1.9821 ± 0.0002
|*a*_iso_^Ti^|	17.5 ± 0.2	
*a*_iso_^H^	−18.16 ± 0.10	
*T* ^H^	[−10.16, −0.67, +10.82] ± 0.20	
[*α*, *β*, *γ*]^H^	[40, 70, 90]° ± 10°	
*a*_iso_^Al^	3.6 ± 1.0	6.2 ± 0.3
*T* ^Al^	[−1.7, +4.3, −2.7] ± 1.0	
[*α*, *β*, *γ*]^Al^	[0, 0, 10]° ± 5°	
*e* ^2^ *qQ*/*h*^Al^	18 ± 2	

### H Q-band ENDOR

The ^1^H hyperfine interaction is no longer resolved in the solid state Q-band CW- and ESE-EPR spectra due to inhomogeneous line broadening. In order to recover such vital information for the structural characterisation of Ti-H, field-dependent Q-band Davies ENDOR spectra were collected at the three canonical positions of the EPR absorption envelope at 20 K, these are indicated by arrows in Fig. 2b. ENDOR spectroscopy provides the NMR spectrum of a paramagnetic centre. Pairs of transitions are expected at *ν*_ENDOR_ = |*ν*_I_ ± 1/2*a*|, where *ν*_I_ is the Larmor frequency of the magnetic isotope under investigation and *a* is the (orientation-dependent) sought hyperfine coupling. At 33.7 GHz, with an applied field of ∼1200 mT ENDOR lines belonging to protons with *a* < 2*ν*_H_, are expected to be centred at ∼51 MHz.

**Fig. 3:**
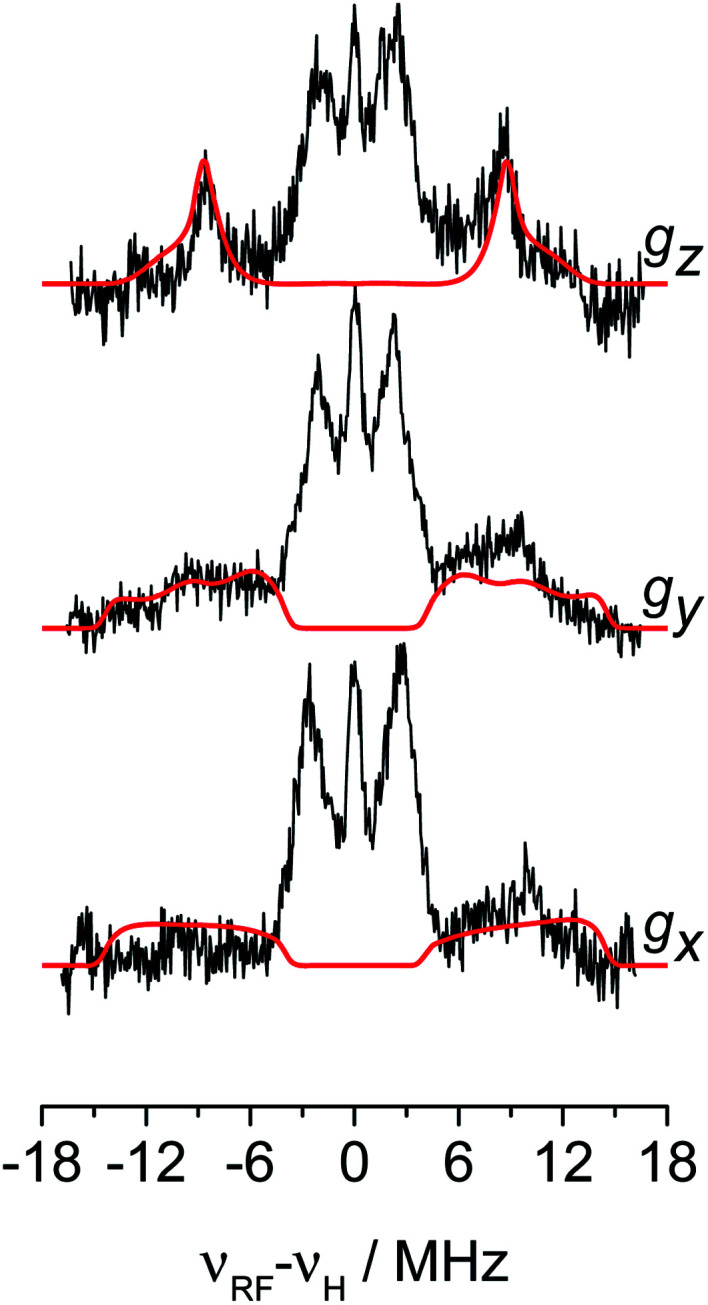
^1^H orientation selective Davies ENDOR spectra of Ti-H recorded at Q-band and at the three canonical positions of the EPR spectrum (black traces) and their simulations (red traces) considering only the hydride ligand with **A**^1^H = [−8, −17.5, −29] and Euler angles = [40, 70, 90]°. Experimental parameters: temperature = 20 K, mw_frequency_ = 33.7 GHz, π pulse = 60 ns, π/2 pulse = 30 ns, *τ* = 400 ns, RF pulse 14 μs. In the *x*-axis the frequency scale gives the deviation of the resonance lines from the Larmor frequency, *ν*_H_, at each field position.

The experimental spectra and their relative simulations are collected in Fig. 3. Two sets of lines are readily discriminated: the outer features centred at about ±9 MHz are attributed to the ^1^H giving rise to the doublet of lines in solution; whereas inner features corresponding to interacting protons with a maximum coupling of the order of 8 MHz (±4 MHz) can be assigned to the protons of the cyclopentadienyl ring and alkyl groups in MMAO.^45^ The relatively low signal-to-noise ratio of the ENDOR experiments stems from the elusive nature of the Ti-H complex which spontaneously forms in solution at a low steady-state concentration.

#### Strongly coupled ^1^H

The outer region of the ENDOR spectra, corresponding to large hyperfine couplings, can be satisfactorily simulated by one ^1^H hyperfine tensor with components *A*^1^H = [−8, −17.5, −29] MHz, where a negative sign is assumed based on a direct Ti-H bond model, as a result of the through-bond spin polarization mechanism, in analogy with the p_π_ C–H fragment.^44^ The best fit was obtained with a relative orientation of the **A** tensor with respect to the **g** tensor expressed by the Euler angles [*α*, *β*, *γ*] = [40, 70, 90]° ± 10°. A slightly less convincing fitting is achieved for the *g*_*x*_ orientation at which a residual contribution from Ti-R may still be present. To further validate the determination of the principal components of *A*^1^H, the simulation of the ENDOR spectrum obtained as the sum of the spectra reported in Fig. 3 was also evaluated (Fig. S3 in ESI†). By removing the orientation selectivity, this procedure simplifies the ENDOR spectrum and allows to assess the overall lineshape of the ENDOR envelope.^46^

The ^1^H hyperfine tensor can be decomposed into an isotropic component *a*_iso_ = −18.16 MHz, fully consistent with the value determined from CW EPR, and an anisotropic – almost fully rhombic – component **T**^1^H = [−10.16, −0.67, 10.82] MHz.

In general, since the anisotropic component of the hyperfine tensor depends on the through space electron dipole–nuclear dipole interaction it can be used to derive bond length and relative orientation between the spin bearing centre (in the present case Ti(iii)) and ^1^H. For short distances (<2.5 Å), as in the case of a nucleus directly bound to the paramagnetic metal ion, the commonly used point-dipole approximation fails and is of no meaningful use. Moreover, the point-dipole approximation would predict an axial anisotropic tensor [*T*, *T*, −2*T*], which is in open contradiction with the experimentally derived (fully) rhombic tensor. A three-point dipole model has been put forward to account for the origin of ^1^H hyperfine anisotropy for a hydride bound to a metal ion with *n*d_*z*^2^_ ground state. The model, derived as an extension of the case of p_π_ C–H fragment,^44^ has been validated by successfully reproducing the anisotropies of the Ni–C state of the [NiFe] regulatory hydrogenase of *Ralstonia eutropha*^1^ and to the Ti(iii) complex Cp_2_TiH_3_AlCTMS_3_ (TMS = Si(CH_3_)_3_; Cp = μ^5^-C_5_H_5_).^42^ By applying the same model to Ti-H (Scheme 1) and by setting the angle between *g*_*z*_ and the Ti⋯H vector equal to the Euler angle *β*, considering a spin density on the Ti 3d_*z*^2^_ orbital of 0.95, the displacement of the 3d_*z*^2^_ lobes equal to 1.40 Å and a Ti⋯H bond length of *r* = 1.8 ± 0.1 Å, the experimental rhombicity derived by ENDOR spectroscopy can be reasonably reproduced *T*_calc_ = [−10.4, −5.7, 16.1] MHz, providing an independent validation of the hyperfine components obtained by fitting.

#### Weakly coupled ^1^H

The central region of the ENDOR spectra shows a number of unresolved proton hyperfine couplings spanning the ∼0 to ±4 MHz. The sharp central peak at *ν*_I_ is the matrix signal due to distant solvent protons. The two wings spanning the regions ∼±2–4 MHz are consistent with the hyperfine couplings of the Cp ring protons at ∼3 Å from Ti, as reported in previous ENDOR studies on the Ti(iii) pseudo sandwich compound CpTicot.^45^ Moreover ^1^H hyperfine couplings of the order of 4.2 MHz were observed in the case of CpTiMe_2_ and attributed to directly ligated Ti(iii)–CH_3_ moieties^41^ (Fig. S3 in the ESI†).

To summarise, since ENDOR peaks depend on the nuclear Larmor frequency, the present experiments unequivocally prove that the doublet signal originates from a ^1^H hyperfine coupling. Furthermore, analysis of the isotropic and anisotropic components and comparison with literature data confirm that the Ti(iii) ion is coordinated by a single hydride ligand placed at ∼1.8 Å and at an angle of ∼70° with respect to the cylindrical axis of the d_*z*^2^_ orbital and that the hydride ligand bears 1.3% of the total spin density. Additional signals are observed and are consistent with the Cp protons at about 3 Å from the Ti centre and further alkyl groups in close proximity (Scheme 1 and Fig. S3 in the ESI†).

### ^27^Al Q-band HYSCORE

**Fig. 4:**
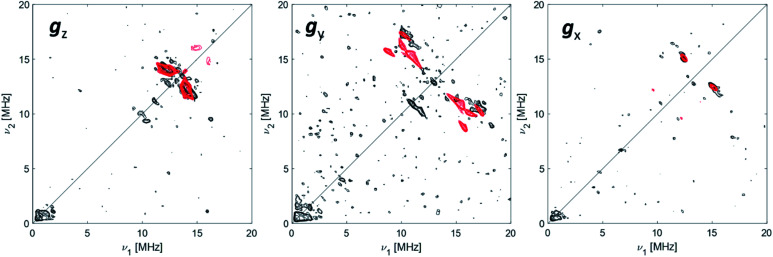
^27^Al orientation selective Q-band HYSCORE spectra (black traces) of Ti-H at the three canonical positions of the EPR spectrum (as indicated by arrows in Fig. 2). The simulations (red traces) correspond to a |A|^27^Al = [2.0, 8.0, 1.0] MHz with Euler angles = [0, 0, 10]° and nuclear quadrupole parameters *e*^2^*Qq*/*h* = 18 MHz, *η* = 0 with Euler angles = [0, 0, 0]°. Experimental conditions: temperature = 50 K, mw_frequency_ = 33.7 GHz, π/2 pulse = 16 ns, π pulse = 32 ns. Both experimental and simulated spectra are the sum of two spectra recorded with two different *τ* values, which were summed together after Fourier transform. The following interpulse delays were used: *τ* = 100 and 150 ns (*g*_*z*_), *τ* = 100 and 148 ns (*g*_*y*_) and *τ* = 110 and 148 ns (*g*_*x*_).

The data collected through CW EPR at X- and Q-bands and Davies ENDOR experiments support that two of the four coordinating sites surrounding the Ti(iii) ion are occupied by a hydride and the Cp ligand, respectively. In order to elucidate the nature of the remaining sites, whether the chlorine ligands of the CpTiCl_3_ precursor are still bound to the central metal and to understand the fate of the MMAO fragment, Q-band HYSCORE spectra were recorded at the three canonical positions of the EPR envelope (indicated by arrows in Fig. 2b). HYSCORE spectra present correlation peaks only in the (+,+) quadrant centred at approximately the ^27^Al nuclear Larmor frequency, indicating the presence of nearby Al nuclei (Fig. 4 and full spectra in Fig. S4 in the ESI†). The maximum coupling of about 8 MHz is observed at a magnetic field position corresponding to the *g*_*y*_ tensor component. Computer simulation (red trace in Fig. 4) indicates that the experimental pattern can be satisfactorily reproduced with a slightly rhombic hyperfine tensor *A*^27^_Al_ = [2.0, 8.0, 1.0] ± 1.0 MHz with the Euler angles [*α*, *β*, *γ*] = [0, 0, 10]° ± 5° in the **g** tensor reference frame and a relatively large nuclear quadrupole coupling (*e*^2^*qQ*/*h* = 18 ± 2 MHz).

The latter value is in line with values reported for Ti(iii)–Al heterobimetallic complexes.^42^ From the isotropic part of the hyperfine coupling *a*_iso_^Al^ = 1/3(*A*_*x*_ + *A*_*y*_ + *A*_*z*_) = 3.6 ± 1.0 MHz and considering the value of *a*_0_ = 3367.76 MHz for unit spin density in the ^27^Al 3s orbital, one can calculate the electron spin population in the Al 3s orbital to be 0.1%. From the anisotropic hyperfine tensor, a lower limit for the Ti–Al distance of about 2 Å can be estimated (Scheme 1a). Both *a*_iso_ and the dipolar matrix correlate with values observed in the case of VO^2+^–O–Al linkages in V doped aluminumphosphate^47^ and Ti^3+^–O–Al linkages in Ti doped aluminosilicate materials.^48^ Somewhat larger values have been reported for Ti^3+^ ions coordinated to an anionic aluminum hydride (alanate) ligand^42^ and for Ti^3+^ ions on the surface of chlorinated aluminum oxide.^49^ Finally, we note that no evidence of Cl interactions could be detected. Chlorine has two magnetically active isotopes ^35^Cl and ^37^Cl both with nuclear spin *I* = 3/2 and relative natural abundance of 75.77% and 24.24%, respectively. However, the HYSCORE spectra presented herein (see also Fig. S4 in the ESI†) lack any sign of magnetic coupling between the electron spin and the pristine chloride ligands, suggesting that they have been removed from the first coordination sphere of Ti. This is in stark contrast with heterogeneous Ziegler–Natta catalysts^50^ and Ti(iii) molecular complexes,^51^ where intense and well defined Cl cross peaks demonstrated the direct Ti–Cl bonding interactions.

### Dynamic properties of Ti-H

The **A**^1^H tensor derived from Q-band ENDOR measurements (Fig. 3), along with the **g** tensor obtained from the simulations of Q-band CW of the frozen solution spectra (Fig. 2a), was used to simulate the motionally averaged (fluid solution) CW spectrum at X-band, assuming an isotropic tumbling.

Considering an intrinsic linewidth 0.3 mT (≈8.4 MHz), which is compatible with the hyperfine values for weakly coupled protons (see Fig. S3 in ESI†) and *a*^Al^_iso_ derived in this work, a good fit is achieved with a rotational diffusion coefficient *D*_r_ = 4 × 10^8^ s^−1^ (see Fig. S5 in ESI†). We note that the successful simulation of the motionally averaged spectrum permits to validate at once the *g*-components and the hyperfine parameters derived in the previous sections. Moreover, through the Stoke–Einstein–Debye equation *D*_r_ can be translated into a hydrodynamic radius (*r*_h_) of ≈16 Å (for an estimation on the uncertainty on *τ*_corr_ and *r*_h_ see Fig. S5 in ESI†). Given that the calculated radius is orders of magnitude larger than what it would be expected for a small metal complex, this analysis further supports the idea that the Ti-H fragment is ion paired to a negatively charged MMAO anion. The estimated radius is consistent with NMR derived values for diamagnetic Ti(iv) complexes forming ion pairs with modified MAO.^52^ From a methodological perspective, this approach provides thus a viable way of estimating hydrodynamic parameters for self-assembled catalytically-relevant paramagnetic complexes, which are not accessible through the more common NMR techniques (*e.g.* PGSE).

### Electronic and geometrical structure of Ti-H

The EPR data presented show thus that activation of CpTiCl_3_ with MMAO in toluene leads to the formation of a structurally and electronically well-defined Ti-H complex, featuring a Ti centred SOMO with dominant d_*z*^2^_ character. ^1^H Davies ENDOR experiments allow the obtainment of compelling evidence for a ^1^H hyperfine coupling characterized by *a*_iso_ = −18.16 MHz and a dipolar tensor **T** = [−10.16, −0.67, 10.82] MHz. The experimental ^1^H dipolar tensor can be satisfactorily reproduced adopting a three-point dipole model^42,44^ with a Ti-H distance of the order of *r* = 1.8 ± 0.1 Å and a tilting angle of ∼70° with respect to the cylindrical axis of the d_*z*^2^_ orbital. M–H distances in early transition metals of the first row range typically in the interval 1.55–1.77 Å,^59^ while longer distances are usually observed for bridging hydride species. A Ti-H distance of 1.84 Å has been reported for a terminal Ti(iv) hydride.^59*b*^

The *a*_iso_ value of open-shell metal hydrides critically depends on both M–H distance and orientation, reflecting the degree of overlap between the metal and the hydride orbitals and it is found to span a wide interval ranging from 3.5 up to 132 MHz, as reported in Table 2 for selected examples. Although no simple correlation can be established, *a*_iso_ values of the order of 18.5 MHz, similar to those measured here correlate with M–H distances of the order of 1.8 Å.

**Table 2:** EPR and structural parameters of selected open-shell metal hydride species

Compound name	Metal	*S*	M–H distance (Å)	*a*_iso_ (MHz)	*T* (MHz)	Reference
[NiFe] hydrogenase *R. eutropha* Ni–C state	Ni(iii)–Fe(ii)ls	1/2		−3.5	[21.9, −7.3, −14.5]	1
α-70^Ile^ MoFe (**2H**)		1/2		24.3	[−13.3, 0.7, 12.7]	2
				22.3	[10.7, −12.3, 1.7]	
Cp_2_TiH_3_AlCTMS_3_ (**2 equivalent H**)	Ti(iii)	1/2	1.892	−9.7	[16.5, −5.5, −11.0]	42
[L^*t*Bu,iPr2^Fe**H**]^−^	Fe(i)	3/2	1.76(8) (X-ray)	−18.5	[28.9, −10.7, −16.1]	53
			1.85 (EPR)			
{[PhBP_3_]Fe}_2_(**μ-H**)(μ-NH)	Mixed valence Fe(ii)–Fe(iii)	1/2	1.68(3) (Fe1, X-ray)	−38.9	[+19.4, −17.4, −2]	54
			1.64(3) (Fe2, X-ray)			
Fe^III^(**H**)(N_2_)(thiolate)	Fe(iii)	1/2	1.54(4)	±43	±[−28, 13, 15]	55
{N_2_–Fe(**μ-H**)_2_Fe–N_2_}^+^	Mixed valence Fe(ii)–Fe(i)	1/2	1.73 (average distance)	−73	[11, −7, −4]	56
Cp2Ti(**μ-H**)MgR (**2 equivalent H**)	Ti(iii)	1/2		−19.6	—	57
Co(CO)_4_**H**^−^	Co(0)	1/2		−132	[2.2, −1.1, −1.1]	58
Ti-**H**	Ti(iii)	1/2	1.8 (EPR)	−18.16	[−10.16, −0.67, 10.82]	This work

In the case of Ti(iii)–Al linked by two μ-H bridging hydrides with a Ti(iii)-H distance of 1.892 Å, ^1^H couplings with *a*_iso_ = 9.7 MHz were observed accompanied by a ^27^Al isotropic hyperfine coupling *a*_iso_ = 9.4 MHz.^42^ Comparison to our values shows that our system is characterized by a larger ^1^H and a smaller ^27^Al hyperfine coupling, implying a reduced spin density on the Al and a larger spin density at the proton. This fact suggests a stronger Ti-H interaction with respect to bridging H.^42^ Even though we cannot definitely exclude an H–Al interaction, this would be rather loose, and all evidence point toward a terminal character of the Ti-H hydride. On the other hand, the *a*_iso_ and *T* values extracted from the analysis of the ^27^Al HYSCORE experiments imply a Ti-L-Al linkage and an intimate interaction between the alumoxane and the transition metal.

It is generally accepted that the MAO activation of group 4 metallocenes proceeds through alkylation steps, which occurs *via* halide/alkyl exchange equilibria possibly up to complete substitution.^34^ In this work no evidence of Cl ions coordination to titanium is obtained pointing to methyl or alkyl bridges, even though oxygen coordination cannot be definitely excluded.^14,18^ Recent experimental and theoretical studies^17,60,61^ on the activation of transition metal catalysts with MAO proved that the strong Lewis acid [AlMe_2_]^+^ cation, resulting from the heterolytic dissociation of AlMe_3_ coordinated to the surface of the MAO cage, is responsible for the *in situ* generation of the alkylmetallocenium cation from the neutral dialkylmetallocene. The resulting MAO anions can in turn coordinate the metallocenium cation *via* methyl bridged structures.^16^ Our EPR results are consistent with this picture pointing to the formation of an ion pair between a MMAO anion linked *via* methyl bridges to a highly unsaturated [CpTi(iii)H]^+^ cation where the H^−^ ligand occupies a terminal position (Scheme 1b). This is at odds with a number of heterobimetallic hydride complexes used in C–H activation reactions, where a bridged TM-H-M motif (TM = transition metal, M = Al, Ga, Zn) has been established through crystallographic data.^9,62^ Finally, from the analysis of the motionally averaged EPR spectrum and the derived rotational diffusion coefficient, an estimate can be made of the hydrodynamic radius of the paramagnetic complex, *r*_h_ ≈ 16 Å, which directly points to a MMAO supported species (ESI Fig. S5†).

**Scheme 2:**
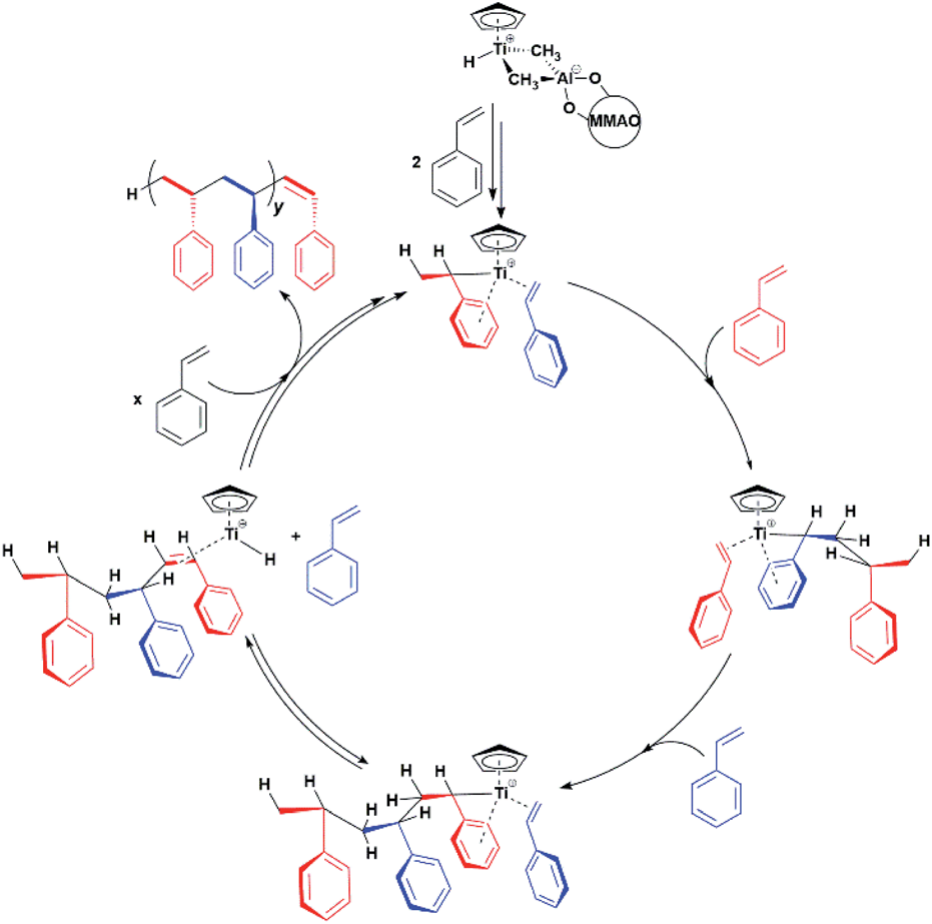
Proposed Ti-H catalytic cycle in the syndiospecific polymerization of styrene.

Our results provide thus the first electronic and structural determination of the labile Ti-H species. They demonstrate that Ti-H is active in syndiospecific styrene polymerization and give further confirmation that the oxidation state of the titanium catalyst is +3, a paramagnetic state. A catalytic cycle involving the Ti(iii)-H species can be proposed and is reported in Scheme 2. The active Ti-H species spontaneously forms upon MMAO activation (Scheme 2 top) and exhibits the characteristic two-line EPR spectrum. In presence of styrene, Ti-H undergoes monomer insertion and chain growth, where both stages are associated with the decrease of the two-line EPR signal (Fig. 1d). β-hydrogen elimination then restores the Ti-H species (Fig. 1e). This latter process may represent either a resting state or an intermediate in equilibrium with the chain growth step.

## Conclusions

In this work, we report a thorough EPR characterization of the organometallic complex obtained by reaction of CpTiCl_3_ with MMAO. A combination of CW and pulse EPR/ENDOR experiments provides compelling evidence for a structurally well-defined open-shell Ti(iii) with 3d_*z*^2^_ ground state featuring a characteristic Ti-H bond and an intimate interaction between the alumoxane and the transition metal. ENDOR and HYSCORE spectra offer unprecedented detail on the bonding interaction of the metal centre with the hydride ligand in the first coordination sphere and an Al ion on the second coordination sphere. This data can be combined to yield a structural model as the one reported in Scheme 1b pointing towards an ion pair between an unsaturated [CpTi(iii)H]^+^ cation and a MMAO anion, with no participation of chloride ligands. It is worth noting that catalytically competent metal-hydrides are usually reactive and difficult to isolate, in this respect EPR spectroscopy and the collection of related techniques provide powerful means to define structural models. To the best of our knowledge, this is one of the few reports of advanced EPR spectroscopy applied to a labile terminal Ti-hydride complex with a well-defined catalytic function. The data presented also establish a wealth of constraints that shall guide future calculations on the reaction pathways and intermediates of the homogenous Ziegler–Natta reaction catalysed by half-titanocenes. The approach described here, with mention to the determination of dynamic parameters, may be of general applicability for structural determination of paramagnetic ion pairs.

## Experimental

### Materials

All manipulations of air- and/or moisture-sensitive compounds were performed under inert atmosphere (dinitrogen or argon) using standard Schlenk techniques or a MBraun drybox. MMAO-3A (7% w/w heptane solution; approximate molecular formula [(CH_3_)_0.7_(i-C_4_H_9_)_0.3_AlO]_*n*_ and CpTiCl_3_ were purchased from Nouryon and Merck, respectively and used without further purification. Commercial grade toluene (Merck) was dried over anhydrous CaCl_2_, refluxed 48 h over Na, and distilled before use. Styrene (Merck) was stirred overnight over CaH_2_, distilled *in vacuum* and stored in the refrigerator prior to use.

### EPR samples preparation

CpTiCl_3_ (65 mg; 300 μmole) was dissolved in toluene (10 mL); 0.10 mL of this stock solution were transferred into a glass vial and premixed with 0.40 mL of the commercial solution of MMAO to produce a toluene solution of [Ti] = 6.0 mM (Al/Ti molar ratio = 250) which was finally introduced into the EPR tube and rapidly analysed (after 5–10 min at room temperature). In the polymerization test, 0.30 mL of styrene were added in a vial containing said solution and rapidly transferred into the EPR tube and analysed.

### EPR spectroscopy

All measurements were collected on a Bruker ElexSys E500 spectrometer operating either a X- or Q-band (∼9.5 and ∼34 GHz respectively). Further details for each set of experiments are given below.

### CW-EPR spectroscopy

Room temperature X-band CW EPR spectra were with a super-high Q resonator (ER4122 SHQE, operating at ∼9.8 GHz). Low temperature Q-band CW EPR spectra were collected using the EN 5107D2 Bruker resonator and an Oxford CF935 cryostat.

### Q-band pulsed spectroscopy

All low temperature Q-band pulsed EPR experiments were collected using the Bruker EN 5107D2 resonator housed in an Oxford CF935 cryostat and a 10 W solid-state microwave amplifier.

Electron-spin-echo (ESE) detected EPR experiments were carried out in the range 20–50 K with the pulse sequence: π/2–*τ*–π–*τ*-echo, with microwave pulse lengths π/2 = 16 ns and π = 32 ns and a *τ* value of 200 ns.

ENDOR measurements were performed at 20 K by employing the Davies pulse sequence (π-RF–π/2–τ–π–τ-echo), with π/2 = 30 ns, π = 60 ns and an RF pulse π_RF_ = 14 μs. ENDOR spectra were collected in stochastic mode by randomly hopping the RF excitation frequency over a 36 MHz sweep with a resolution of 0.07 MHz (512 points).

HYSCORE spectra were recorded at 50 K using the pulse sequence π/2–*τ*–π/2–*t*_1_–π_inv_–*t*_2_–π/2–*τ*-echo. The pulse length for inversion pulse (π_inv_) and the π/2 and π pulses were 28 ns 16 and 32 ns, respectively. The first interpulse delay, *τ*, was adjusted at each field position either to maximise or minimise the ^27^Al Larmor contribution. Eight-step phase cycling was used to eliminate unwanted echoes. The experimental dataset comprised a 300 by 300 points. Time-domain spectra were baseline-corrected (third-order polynomial), apodized with a hamming window function, zero-filled to 2048 points, and fast Fourier-transformed to yield the frequency-domain spectra.

Both ENDOR and HYSCORE spectra were recorded at all three canonical positions to fully characterise the angular dependence of the coupling tensors.

Further spectrometer and experimental settings are given in the corresponding figure captions.

### EPR simulations

Simulations of both CW and PULSED EPR spectra were performed using EasySpin 5.2.28 toolbox^63^ within the Matlab 2019b software suite (The Mathworks Inc., Natick, MA).

### Conversion between magnetic field and frequency units

Much of the existing literature on Ti(iii) complexes involved in Ziegler–Natta catalysis reports the hyperfine couplings in unit of Gauss (G). This unit of measurement is not a SI unit and its use is discouraged in favour of Tesla (T) or, more commonly milli-Tesla (mT). However, given that hyperfine couplings (*a*) express an interaction energy, Mega-Hertz (MHz) should be used. Useful conversion factors are: 1 mT = 10 G (*i.e.* 1 T = 10 000 G) and 
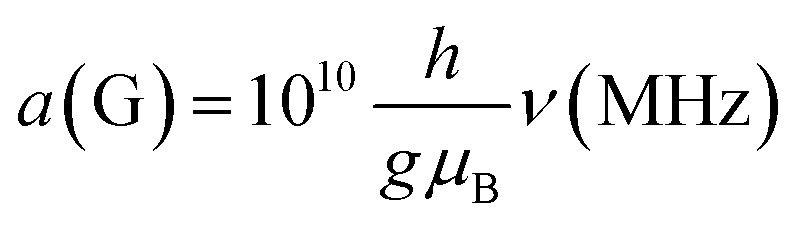
. It is worth noting that the conversion between G and MHz is inversely proportional to the *g*-factor of the species considered.

## Conflicts of interest

Authors declare no conflicts of interest.

## Supplementary Material

SC-011-D0SC04967K-s001
