# Supplementary material for: Structure and dynamics of catalytically competent but labile paramagnetic metal-hydrides: the Ti(iii)-H in homogeneous olefin polymerization
Source: Chem Sci. 2020 Sep 24;11(46):12436–45. doi: 10.1039/d0sc04967k (PMC8162776; doi:10.1039/d0sc04967k)
Supplement: SC-011-D0SC04967K-s001 [file SC-011-D0SC04967K-s001.pdf]

Electronic Supplementary Material (ESI) for Chemical Science.
This journal is © The Royal Society of Chemistry 2020

Supporting information

Structure and Dynamics of Catalytically Competent but Labile Paramagnetic Metal-hydrides: the Ti(III)-H in Homogeneous Olefin Polymerization

Enrico Salvadori[a], Mario Chiesa*[a], Antonio Buonerba[b], Alfonso Grassi[b]

a Dr E. Salvadori, Prof M. Chiesa
Department of Chemistry, University of Turin, Via Pietro Giuria 7, Torino 10125, Italy
E-mail: mario.chiesa@unito.it

b Dr A. Buonerba, Prof A. Grassi
Dipartimento di Chimica e Biologia, Università degli Studi di Salerno,Via Giovanni Paolo II, 132, I-84084 Fisciano, SA, Italy

Table of contents

Structures of MAO and MMAO 2
Calculation of *s*-spin density from the isotropic hyperfine constant 3
Individual contributions of Ti-H and Ti-R 4
ENDOR simulation inclusive of weakly coupled protons 5
Additional HYSCORE spectra 6
Dynamic properties of Ti-H 7
Supporting Information References 8

Structures of MAO and MMAO

Methylaluminoxane (MAO) is an alkyl aluminium compound used to activate Ziegler-Natta homogenous catalysts via alkylation of the transition metal ion. Further, MAO acts as a Lewis acid to stabilize the formed adduct establishing an ion-pair. However, a definitive consensus about MAO exact chemical structure has not yet been reached despite its central role in olefin polymerization catalysis and the vast literature on the topic. Most recent reports suggest that MAO would consist of a mixture of oligomers - general formula $[-Al(CH_3)O-]_n$ - which produce aggregates of 150- 200 Al atoms terminated at the surface with unreacted $AlMe_3$ (TMA) (see figure S1). MAO's properties can be summarised as follows: i) a general formula $(Me_{1.4-1.5}AlO_{0.75-0.80})_n$; ii) an average molecular weight MW of 1200− 2000 Da; iii) a degree of polymerization n = 20−30; and iv) coordination numbers for Al = 4 and O = 3 (minor Al species with coordination = 3 are also present).

In this picture, the highly reactive $[AlMe_2]^+$ cation, thought to be the relevant Lewis acid capable of producing the group 4 alkylmetallocenium cation active in olefin polyinsertion, would result from the equilibration of linear and cage-like structures

Figure S1 reports three possible coordination - labelled a), b) and c) - of Al at the surface of a MAO cage and the resulting anions after elimination of $[Al(CH_3)_2]^+$.[A]

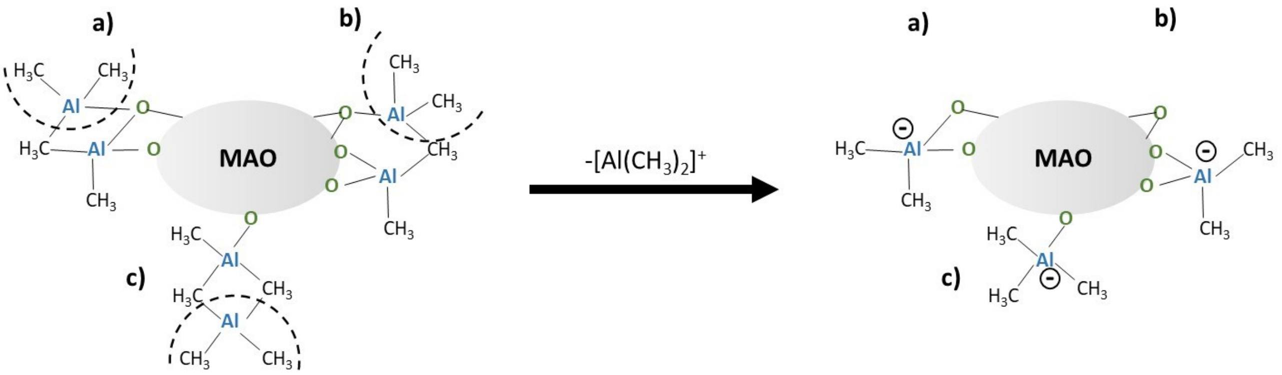

Figure S1: Schematic of MAO sites amenable of $[AlMe_2]^+$ dissociation and resulting MAO anions. Three plausible sites are depicted based on the data reported in reference A.

In modified-MAO (MMAO) some isobutyl groups replace methyl groups in the structure giving an overall composition of $[(CH_3)_{0.7}(i\text{-}C_4H_9)_{0.3}AlO]_n$. In turn, this modification increases stability and solubility in aliphatic hydrocarbon solvents, but without affecting the three-dimensional structure or reactivity.

Calculation of *s*-spin density from the isotropic hyperfine constant

From the isotropic (a_{iso}) hyperfine couplings, the *s*-contribution of each nucleus to the SOMO in a molecular fragment can be obtained. Assuming that the a_{iso} hyperfine coupling interaction at a given nucleus is proportional to the electron spin density at that nucleus, one can obtain the spin population in *s*-type orbitals ρ_s. For an unpaired electron (free electron, g_e=2.0023) with a unitary spin population (ρ_s =1) in an s-type orbital, one would observe an isotropic hyperfine coupling constant of a^0_{1H} = 1420 MHz for a ^1H nucleus and a^0_{27Al} = 3367.76 MHz for a ^{27}Al nucleus. Including a correction for the difference in the *g* values, the spin populations in s-type can thus be estimated as:

$$\rho_s = \frac{a_{iso}\ g_e}{a^0\ g_{iso}}$$

where g_e is the free electron *g*-value, g_e = 2.00231930436256(35).

Individual contributions of Ti-H and Ti-R

The simulations of the CW spectra reported in Figures 1b and 2a (main text) contain both the contribution of Ti-H and Ti-R. In Figure S2 we report the individual spectral components for the X-band spectrum at RT and the Q-band spectrum at 50 K. The relative spin concentrations [Ti-H]:[Ti-R] vary from 30:70 at RT to 35:65 at 50 K as result of the different relaxation properties of the two species. The simulation parameters are those reported in Table 1 (main text).

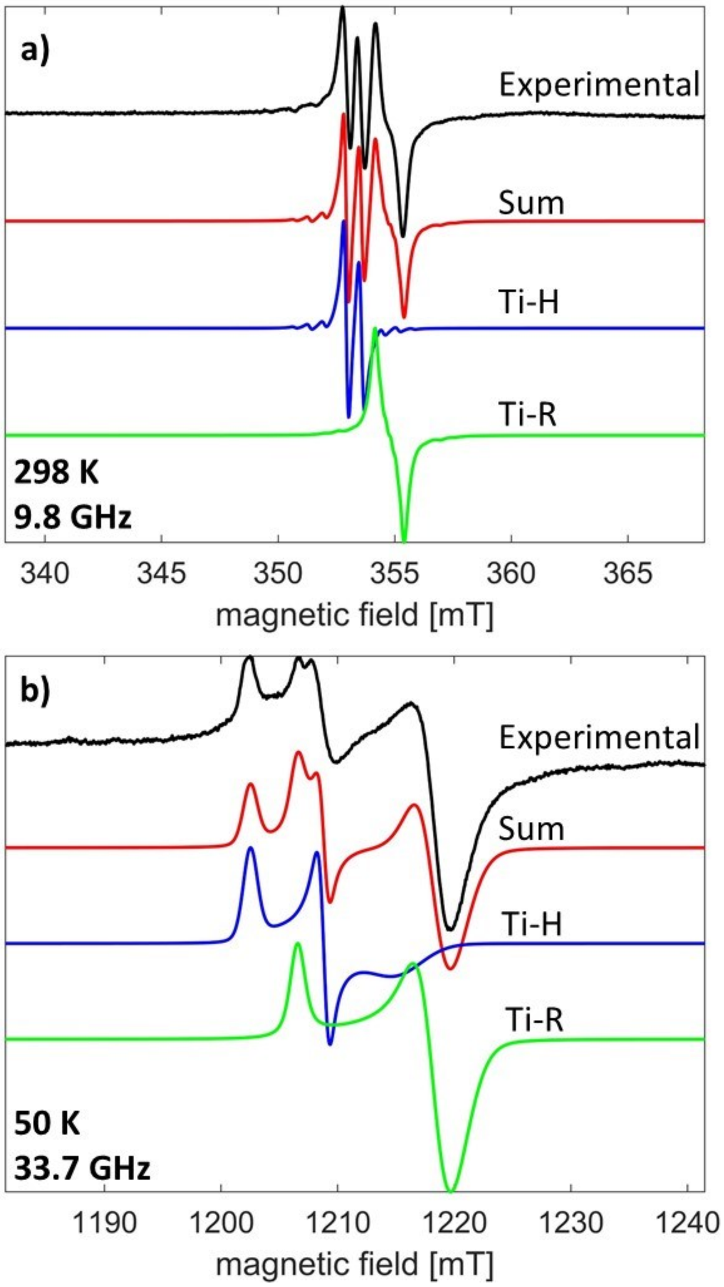

Figure S2: Simulations of Ti-H and Ti-R individual components contributing to the fluid solution X-band spectrum (a) and frozen solution Q-band spectrum (b).

ENDOR simulation inclusive of weakly coupled protons

Figure 3 in the main text reports the orientation selective simulations for the hydride proton. A reasonable simulation of the whole ENDOR spectrum (obtained as the sum of the three spectra presented in Figure 3, main text) can be accomplished considering three distinct contributions: i) the aforementioned hydride protons; ii) typical values for Cp protons[B]; and iii) an additional coupling compatible with those measured for CpTi(III)$(CH_3)_2$[C] and is reported in Figure S4. Given the low signal-to-noise ratio of the ENDOR spectra and to simplify the determination of the principal components of the hyperfine tensor by eliminating the orientation selectivity, the sum of the individual ENDOR spectra were simulated. A similar procedure was described by Enemark[D] and is justified by : 1) the small *g* anisotropy so that its effects on the anisotropy of the hyperfine interaction can be neglected; and 2) the negligible change of ν_I across the EPR spectrum (this also depends on condition 1).

The hyperfine parameters (a_{iso} and T) for each set of protons are reported in the table below. The sum simulation does not include any contribution for remote protons (a ~ 0 MHz), therefore the peak centered at ν_{RF}-ν_H = 0 MHz is not accounted for. This data supports the idea that the interaction between Ti and Al is mediated by the methyl groups of MAO and provide an estimate of the hyperfine couplings as well as corroborating the fit of the hydride proton presented in the main text.

Table S1: Summary of the ENDOR parameters for Ti-H complex. We note that the hyperfine couplings for Cp and CH_3(MMAO) are indicative.

	**a_{iso} (MHz)**	**[Tx,Ty,Tz] (MHz)**
**Hydride**	-18.16	[-10.16, -0.67, +10.82]
**Cp**	+4.06	[-2.56, -2.66, +5.24]
**CH_3 (MMAO)**	+5.60	[-1.40, -1.40, +2.80]

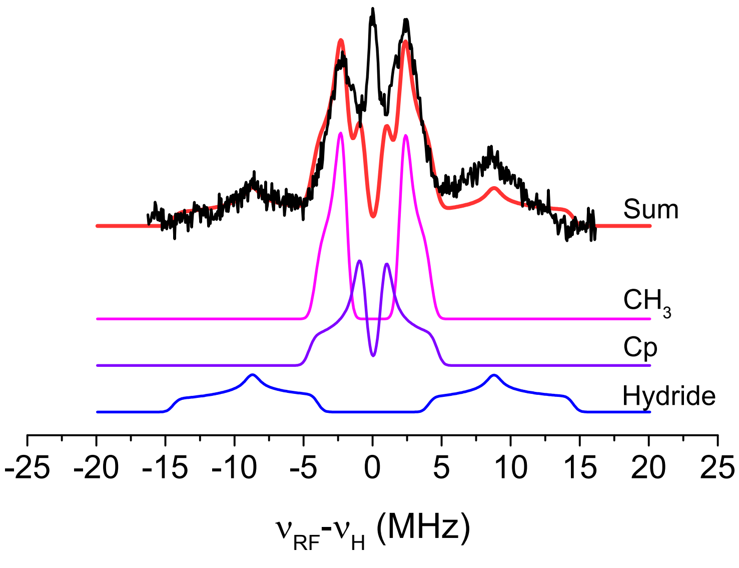

Figure S3: Simulation (red trace) of the sum ENDOR spectrum (sum of the three spectra reported in Figure 3 main text, black trace) including three contributions: i) alkyl protons of MMAO; ii) C protons; and iii) the hydride proton. The relative hyperfine values are reported in Table S1.

Additional HYSCORE spectra

At each field position, HYSCORE spectra (*π/2-τ-π/2-t_1-$π_{inv}$-t_2-π/2-τ-echo*) were recorded with two different τ values to minimise blind spot effects (g_x = 110 and 148 ns, g_y = 100 and 148 ns, g_z = 100 and 150 ns). Here we report the full spectra - both (+,+) and (-,+) quadrants - where the spectra recorded at different τ values were summed together after Fourier transform. The supplementary spectra do not provide any additional constrains for the determination of ^{27}Al parameters, but also do not show any ^{35,37}Cl signals further supporting the notion that the Cl^- ligands present in the parent CpTi(IV)Cl_3 are completely lost upon activation with MMAO in toluene.

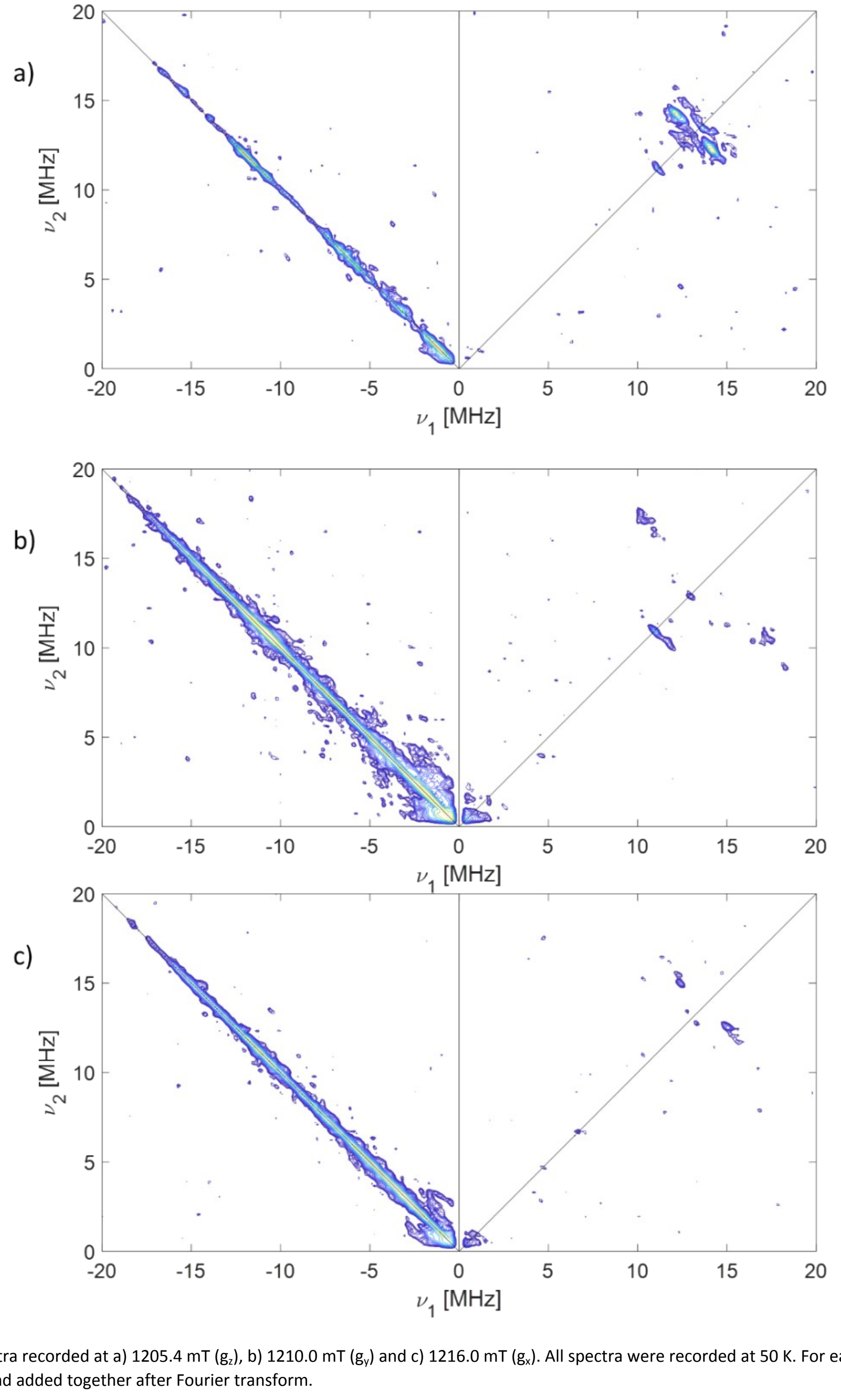

Figure S4. HYSCORE spectra recorded at a) 1205.4 mT (g_z), b) 1210.0 mT (g_y) and c) 1216.0 mT (g_x). All spectra were recorded at 50 K. For each magnetic field setting, two spectra were recorded and added together after Fourier transform.

Dynamic properties of Ti-H

The **A**^{1}H tensor derived from Q-band ENDOR measurements (Figure 3 main text), along with the **g** tensor obtained from the simulations of Q-band CW of the frozen solution spectra (Figure 2a main text), was used to simulate the motionally averaged (fluid solution) CW spectrum at X-band, assuming an isotropic tumbling. Considering an intrinsic linewidth 0.3 mT (≈ 8.4 MHz), which is compatible with the hyperfine values derived in this work, a series of simulations has been performed. Figure S5A reports the whole set of simulated EPR spectra for Ti-H at various correlation times, from τ_{corr} = 1×10^{-12} s (fast motion limit) to τ_{corr} = 5×10^{-09} s. Figure S5B, C, and D reports a direct comparison of the experimental spectrum with the simulated one corresponding to the best fit (**D**, τ_{corr} = 4×10^{-10} s) and the lower un upper limits (**B,** τ_{corr} = 2×10^{-10} s and **C,** τ_{corr} = 6×10^{-10} s, respectively). As a point of reference a direct comparison in the fast motion limit (τ_{corr} = 1×10^{-12} s) is reported in Figure S5E.

The estimated rotational correlation time can be translated into a hydrodynamic radius (r_h) through the Stoke-Einstein-Debye[E,F] equation

$$r_h = \sqrt[3]{\frac{3k_BT}{4\pi\eta D_{corr}}}$$

where η is the dynamic viscosity (for toluene η = 0.00055 N s m^{-2}), k_B is the Boltzmann constant (J K^{-1}), T is the absolute temperature (K) and D_{corr} is related to τ_{corr} derived from spectral simulation through the relation $D_{corr} = 1/6*\tau_{corr}$. Using the τ_{corr} values derived from the analysis of the motionally averaged spectrum, the hydrodynamic radius for the Ti-H/MMAO ion pair in toluene solution is found to be within 1.28 < r_h < 1.85 nm, with a most likely value of 1.61 nm. This value is somewhat larger than what reported for Zr complexes ion-paired to MAO measured through NMR.[G] and consistent with values for Ti complexes forming ion pairs with modified MAO[H].

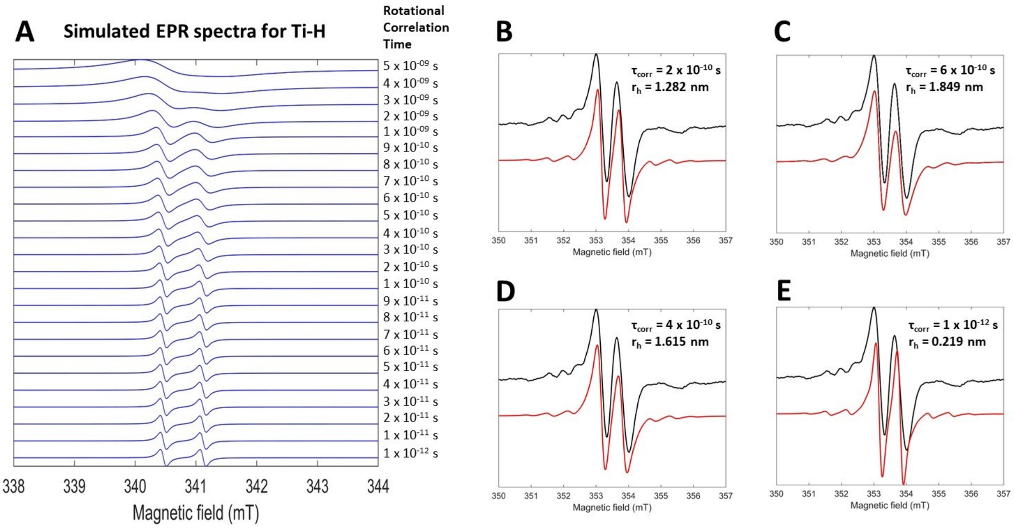

Figure S5: Panel **A** reports the simulated spectra for Ti-H at various rotational correlation times in the interval 1×10^{-12} s < τ_{corr} < 5×10^{-09} s. Simulations were performed at mw$_{frequency}$ = 9. 5 GHz and considering the full **g** and **A**^{1}H tensors, as reported in Table 1 (main text). Panels **B** to **D** report a direct comparison of the experimental spectrum of Ti-H at RT with simulated spectra at various correlation times, namely τ_{corr} = 2×10^{-10} s (lower limit, **B**), 4×10^{-10} s (best fit, **D**), 6×10^{10} s (upper limit, **C**). The corresponding hydrodynamic radii are also reported (1.28 < r_h < 1.85 nm). For reference, panel **E** shows the comparison of the experimental spectrum for Ti-H with the spectrum simulated in the fast motion regime (τ_{corr} = 1×10^{-12} s). The Easyspin routine *Garlic* was used to simulate all spectra reported in Figure S6.

The good fit of the experimental X-band CW spectrum further reinforces the validity of the parameters derived from the EPR/ENDOR analysis and shows how EPR data can be used to infer dynamic properties of paramagnetic ion pairs.

Supporting Information References

[A] H.S. Zijlstra, M. Linnolhati, S. Collins, J.S. McIndoe *Organometallics* 2017, **36**, 1803-1809

[B] D. Gourier, E. Samuel *J. Am. Chem. Soc*. 1987, **109**, 4571-4578

[C] S. Van Doorslaer J. J. Shane, S. Stoll, A. Schweiger, M. Kranenburg, R. J. Meier. *J. Organomet. Chem*. 2001, **634**, 185–192

[D] A.V. Astashkin, E.L. Klein, J.H. Enemark, J. Inorg. Biochem. 2007, **101**, 1623-1629 (and references therein)

[E] I. Peric, D. Merunka, B.L. Bales, M. Peric *J Phys Chem Lett*. 2013, **4**(3), 508–513

[F] M.R. Kurban *J Phys Chem A*. 2013, **117**(7), 1466–1473

[G] D.E. Babushkin, H.H. Britzinger *J. Am. Chem. Soc.* 2002, **124**, 43, 12869-12873

[H] F. Zaccaria, C. Zuccaccia, R. Cipullo. P.H.M. Budzelaar, A. Macchioni, V. Busico, C. Ehm *ACS Catal.* 2019, **9**, 4, 2996-3010
